# On Influenza and Ozone

**Published:** 1849-08

**Authors:** 


### ECLECTIC DEPARTMENT,

### AND SPIRIT OF THE MEDICAL PERIODICAL PRESS.

*On Influenza and Ozone.*—By Dr. Spengler, of Elville.—Dr. Spengler remarks, on the incomplete state of our knowledge of the etiology of epidemic diseases, that the present crude theories of their dependence upon certain indefinite degrees of heat or cold in the weather will no longer be admitted; but that, by following up the discovery of ozone by Schonbein, we shall, having a tangible point whence to start, arrive at the clearness of truth, instead of the darkness which has hitherto hung over the subject.

He states, that in the village of Roggendorf, in Mecklenburgh, towards the close of 1846, slight catarrhal affections became prevalent, that but a slight trace of ozone was then to be detected in the air. With the opening of the following year, however, these catarrhal affections assumed the severest forms of tracheal and bronchial disease, and hooping-cough became common, both among children and adults; then re-agents detected a great increase of ozone in the atmosphere, and, at the same time, influenza spread over the district. On the 9th of January the *ozonometer* showed a still further increase in the proportion of ozone present in the air. On the same day two persons died of influenza, and gradually the influenza spread more extensively, until the 21st scarcely an individual had escaped. Thus there seemed a decided connection between the presence of ozone in the air and the spread of the epidemic.

Ozone is formed in the air by the decomposition of its water through disturbances of its electrical equilibrium; hence the peculiar pungent sulphurous and phosphoric odor. The nature and composition remains as yet uncertain. Sulphuric, probably also telluric and selenic acids, and phosphoric acid destroy it. A very small proportion of the vapors of ether or alcohol, or of olefiant gas, will also prevent its development.

Its best test is iodide of potassium, which will detect its presence in infinitely small quantities in the air. A piece of paper moistened with a mixture of starch and solution of iodide of potassium forms an *ozonometer* far exceeding in delicacy the most accurate galvanometer, or the most sensitive nose. The smallest quantity of free ozone (even only in the proportion of a hundred-thousandth) when neither galvanometer nor eudiometer show any change in the air, will be rendered manifest by the discoloration produced by the free iodine.

At the beginning of the epidemic we have noticed that there was but slight discoloration; it gradually became darker, till at last the ozonometer exhibited a blackish-brown color.

As the presence of ozone in the air is due to its electrical decomposition, it is necessarily influenced by its electrical tension.

If the prevalence of influenza and epidemic catarrh be owing to ozone, the vapors of sulphur, or sulphurous gases must be protective against it. This is confirmed by (while it explains the immunity of) those engaged in or living near sulphur works.

Dr. Spengler has been induced to publish his observations with the hope of inducing others to make further investigations into the existence and nature of ozone.—*Med. Gaz. from Henle's Zeitschrift.*

